# Induction of IgG3 to LPS via Toll-Like Receptor 4 Co-Stimulation

**DOI:** 10.1371/journal.pone.0003509

**Published:** 2008-10-23

**Authors:** Francisco J. Quintana, Aderet Solomon, Irun R. Cohen, Gabriel Nussbaum

**Affiliations:** 1 Center for Neurologic Diseases, Harvard Medical School, Boston, Massachusetts, United States of America; 2 The Department of Immunology, The Weizmann Institute of Science, Rehovot, Israel; 3 Institute of Dental Sciences, Hebrew University-Hadassah School of Dental Medicine, Jerusalem, Israel; University of Miami, United States of America

## Abstract

B-cells integrate antigen-specific signals transduced via the B-cell receptor (BCR) and antigen non-specific co-stimulatory signals provided by cytokines and CD40 ligation in order to produce IgG antibodies. Toll-like receptors (TLRs) also provide co-stimulation, but the requirement for TLRs to generate T-cell independent and T-cell dependent antigen specific antibody responses is debated. Little is known about the role of B-cell expressed TLRs in inducing antigen-specific antibodies to antigens that also activate TLR signaling. We found that mice lacking functional TLR4 or its adaptor molecule MyD88 harbored significantly less IgG3 natural antibodies to LPS, and required higher amounts of LPS to induce anti-LPS IgG3. *In vitro*, BCR and TLR4 signaling synergized, lowering the threshold for production of T-cell independent IgG3 and IL-10. Moreover, BCR and TLR4 directly associate through the transmembrane domain of TLR4. Thus, *in vivo*, BCR/TLR synergism could facilitate the induction of IgG3 antibodies against microbial antigens that engage both innate and adaptive B-cell receptors. Vaccines might exploit BCR/TLR synergism to rapidly induce antigen-specific antibodies before significant T-cell responses arise.

## Introduction

Antibodies perform a broad array of functions dictated by the constant region of their heavy chain. Mature B cells diversify the number of functions performed by a particular antibody by switching from IgM to IgG expression while preserving their antigen specificity [1]. IgG production usually results from the integration of two signals: An antigen specific signal provided via the B-cell antigen receptor (BCR), and co-stimulatory signals provided by T cells and dendritic cells in the form of cytokines and/or membrane-bound ligands [2]. This two signal requirement limits the risk of undesired immunopathology but imposes a 5–7 day delay in the induction of an effective antibody response, a delay that might be far too long to fight fast growing pathogens.

Toll-like receptors (TLRs) and their associated signaling pathways are mostly known for their role in the control of innate immunity [3], but they also contribute to the induction [4] and control [5] of adaptive immunity. TLR signaling participates in the maintenance of B-cell memory in humans [6], and although it has been shown to facilitate switching from IgM to IgG expression [7], [8], [9], it is still unclear to what extent it is required for the induction of antigen-specific antibodies [9], [10], [11], [12]. The importance of establishing the role of TLRs in the control of the humoral response is highlighted by the outcome of vaccination against *B. burgdorferi* in humans: individuals with diminished TLR1/2 function showed a significant decrease in their antibody response to the *B. burgdorferi* OspA protein (a TLR2 ligand) [13]. In the more physiological set up of an infection, the collaboration between TLR and BCR signaling might be important for the early activation of pathogen-specific B cells that help contain the infection until the establishment of a mature T cell response.

LPS produced by Gram-negative bacteria activates B cells by way of innate TLR4 and/or TLR2 signaling pathways [14]. In this work we analyzed the contribution of TLR4 to the induction of mouse IgG antibodies to LPS. We found that IgG antibodies to LPS are induced by the synergistic interaction of low concentrations of LPS with TLR4 and the BCR. This synergism results from the association of BCR and TLR4 molecules in a B-cell membrane complex mediated by the TLR4 transmembrane domain. This mechanism might be therapeutically exploited for the induction of antigen-specific antibodies.

## Results

### Natural IgG antibodies to LPS are not detected in mice lacking a functional TLR4

IgM and IgG antibodies to self and non-self antigens can be induced by immunization, however some antibodies also arise naturally in the absence of known immunization or overt clinical disease; these antibodies have been termed natural antibodies [15]. Natural antibodies against microbial antigens are probably the result of repeated sub-clinical encounters with normal flora and infectious agents, and have been shown to be effective in the control of infection [16], [17], [18], [19].

**Figure 1:**
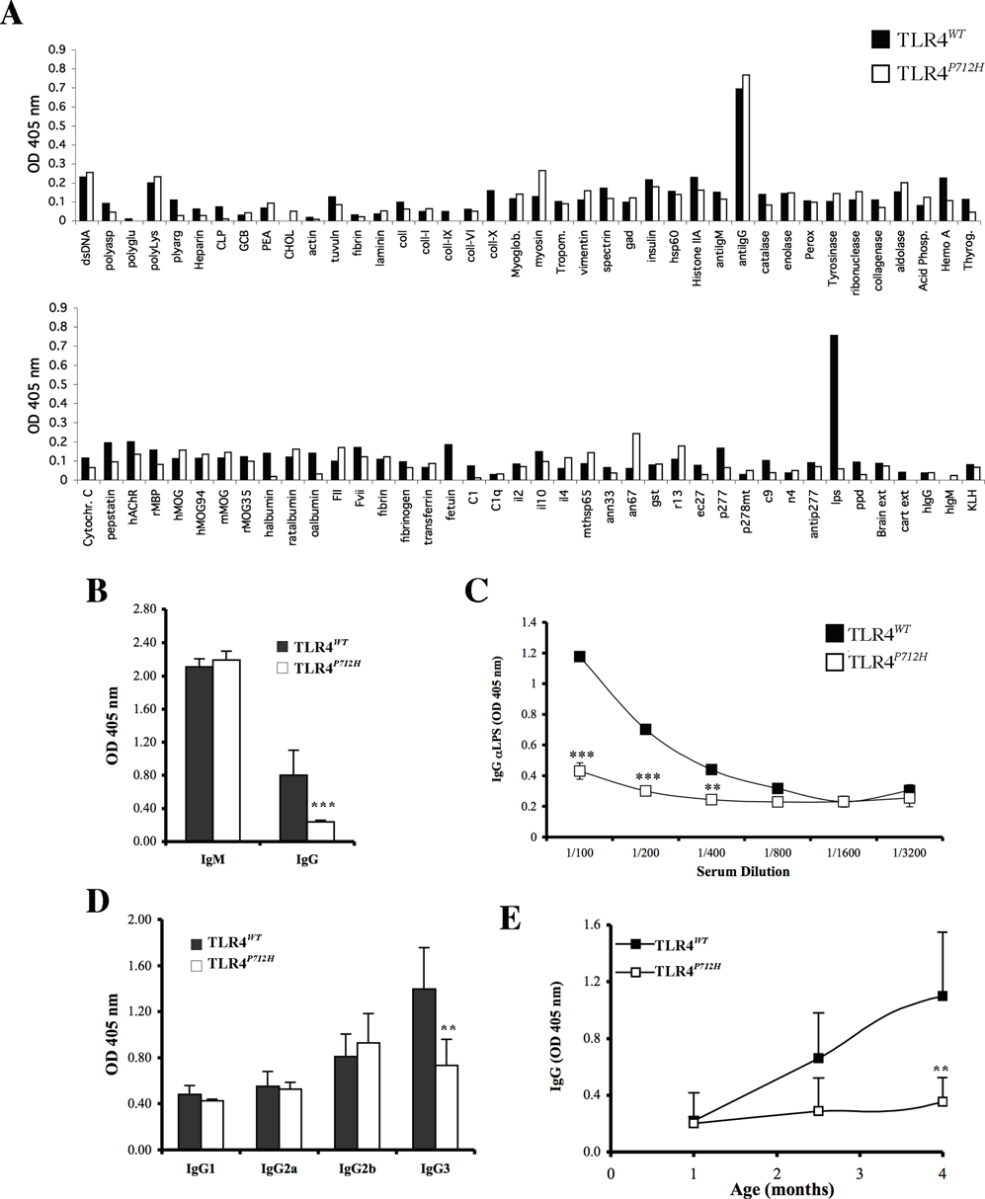
Natural IgG antibodies to LPS are not detectable in TLR4*^P712H^* mice. A. IgG reactivity in pooled blood samples from non-immunized TLR4*^WT^* and TLR4 *^P712H^* mice. B. IgM and IgG antibodies to LPS in individual TLR4*^WT^* and TLR4 *^P712H^* mice (n = 5, *** *P*<0.001 when compared to TLR4*^WT^* mice). C. Titration of spontaneous IgG antibodies to LPS in TLR4*^WT^* and TLR4*^P712H^* mice (n = 5 per group, ** *P*<0.01 and *** *P*<0.001 when compared to TLR4*^WT^* mice). D. IgG subclass of antibodies to LPS in individual TLR4*^WT^* and TLR4 *^P712H^* mice (n = 5, ** *P*<0.01 when compared to TLR4*^WT^* mice). E. Time course of the induction of IgG3 antibodies to LPS (n = 6 per group, ** *P*<0.01 when compared to TLR4*^WT^* mice).

C3H/HeJ mice harbor a P712H point mutation in the TLR4 gene that results in a non-functional protein, whereas other C3H mouse strains express functional TLR4 [20], [21]. We probed the repertoire of natural IgG antibodies in pooled sera from non-immunized 14-week old C3H/HeJ (hereafter TLR4*^P712H^*) or C3HeB/FeJ (hereafter TLR4*^WT^*) mice using a panel of 87 self and non-self antigens (**Table S1**). Both strains of mice harbored similar profiles of natural IgG antibodies except for antibodies to LPS that were present in the sera of TLR4*^WT^* mice, but not detectable in the sera of TLR4*^P712H^* mice (**Figure 1A**). This strain difference was confirmed by testing sera of individual mice (**Figure 1B**). Surprisingly, there were no differences in the levels of anti-LPS IgM between the two strains despite the significant difference in anti-LPS IgG (**Figure 1B**). To rule out a prozone effect, sera were serially diluted and tested for anti-LPS IgG. The TLR4*^P712H^* sera were not reactive to LPS at any dilution (**Figure 1C** **)**. The lack of IgG reactivity to LPS in TLR4*^P712H^* mice was not due to masking of LPS epitopes by IgM because sera were pre-treated with 0.05 M β-mercaptoethanol [22] to disrupt IgM. The natural anti-LPS IgG antibodies were almost exclusively of the IgG3 subclass (**Figure 1D**), and recognized an oxidation sensitive epitope (data not shown), as has been previously described for antibodies to the carbohydrate portion of LPS [23].

We analyzed the appearance of anti-LPS IgG over time (**Figure 1E**). At 4 weeks of age, anti-LPS IgG was not detectable in either strain, however both strains manifested anti-LPS IgM (data not shown). Starting at week 8, anti-LPS IgG was detectable in the sera of the TLR4*^WT^* mice, but not in the sera of the TLR4*^P712H^* mice, through age 16 weeks (**Figure 1E**).

Based on these results, and taking into consideration that LPS is a T cell independent antigen known to elicit primarily IgM and IgG3 antibodies [24], we focused our investigations on the role of TLR4 in the generation of IgG3 to LPS.

### The synthesis of anti-LPS IgG3 involves MyD88-dependant TLR4 signaling

**Figure 2:**
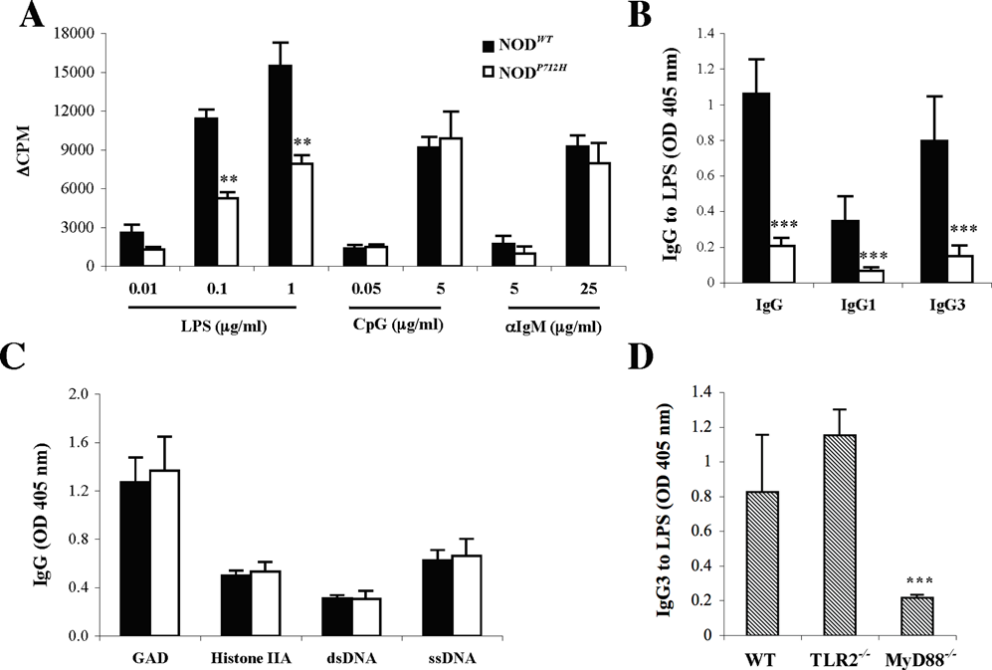
Natural Anti-LPS IgG3 is generated via MyD88-dependant TLR4 signaling. A. Purified B cells from NOD *^P712H^* and NOD*^WT^* mice were activated *in vitro* with LPS, CpG or αIgM and the proliferative response was assayed. ** *P*<0.01 when compared to NOD*^WT^* mice. B. Total IgG and IgG1 vs. IgG3 antibodies to LPS in NOD*^WT^* (n = 13) and NOD *^P712H^* mice (n = 7). *** *P*<0.001 when compared to NOD*^WT^* mice. C. IgG antibodies to GAD, histone IIA, dsDNA and ssDNA in NOD*^WT^* (n = 13) and NOD *^P712H^* mice (n = 7). D. IgG3 antibodies to LPS in WT, TLR2^−/−^ and MyD88^−/−^ mice (n = 4). *** *P*<0.001 when compared to WT or TLR2^−/−^ mice. Results represent mean (±SD) of each group.

To confirm that the difference in anti-LPS IgG was due to the differences in the TLR4 gene and not to other genetic differences between the C3H sub-strains, we back-crossed the TLR4 P712H point mutation from C3H/HeJ mice onto the genome of NOD/LtJ mice that normally bear functional wild-type TLR4 (**Figure S1**). We previously demonstrated that NOD mice develop anti-LPS IgG by 8 weeks of age in the absence of immunization, just as do C3H mice homozygous for wild-type TLR4 genes [25]. Since NOD mice develop a rich network of natural autoantibodies [25], we could test the impact that loss of TLR4 signaling has upon these reactivities. Breeding the mutant TLR4 allele into the NOD/LtJ genome (hereafter NOD*^P712H^*) specifically impaired their B-cell proliferative response to LPS, whereas proliferation induced by a mitogenic antibody to surface IgM (αIgM), or by a CpG oligonucleotide (known to activate murine B-cells via TLR9 [26]) remained intact (**Figure 2A**). The NOD*^P712H^* and littermates bearing WT TLR4 (hereafter NOD*^WT^*) all developed anti-LPS IgM antibodies (data not shown), but only the NOD*^WT^* mice developed anti-LPS IgG: IgG3 and to a lower extent, IgG1 (**Figure 2B**). Regardless of the functionality of their TLR4 both the NOD*^P712H^* and the NOD*^WT^* mice spontaneously developed IgG antibodies that bound the self-antigens GAD, Histone IIA, dsDNA and ssDNA (**Figure 2C**), among others. Thus although both strains were equally exposed to LPS, as suggested by the similar levels of LPS-reactive IgM, functional TLR4 is specifically needed for the spontaneous development of natural anti-LPS IgG antibodies.

TLR2 governs the innate response to LPS from gram-negative organisms such as *P. gingivalis* (pgLPS) and to lipoproteins and peptidoglycans [3]. Both TLR2- and TLR4-dependent signaling pathways operate through the adaptor molecule MyD88 [3], [14], although TLR4 signaling can proceed through a MyD88-independent pathway [14]. To confirm the specificity of the control by TLR4 of the induction of anti-LPS IgG3 antibodies we followed the induction of natural IgG3 antibodies to LPS in TLR2-deficient mice: no difference was seen in their levels when compared to matched controls (**Figure 2D**). However, mice deficient in the intracellular adaptor protein MyD88 failed to develop anti-LPS IgG3 (**Figure 2D**), although they did develop natural anti-LPS IgM (data not shown). Thus, the appearance of IgG3 LPS-specific antibodies is TLR2 independent and is controlled by TLR4 via the MyD88 pathway.

### TLR4 controls the response to LPS upon immunization

**Figure 3:**
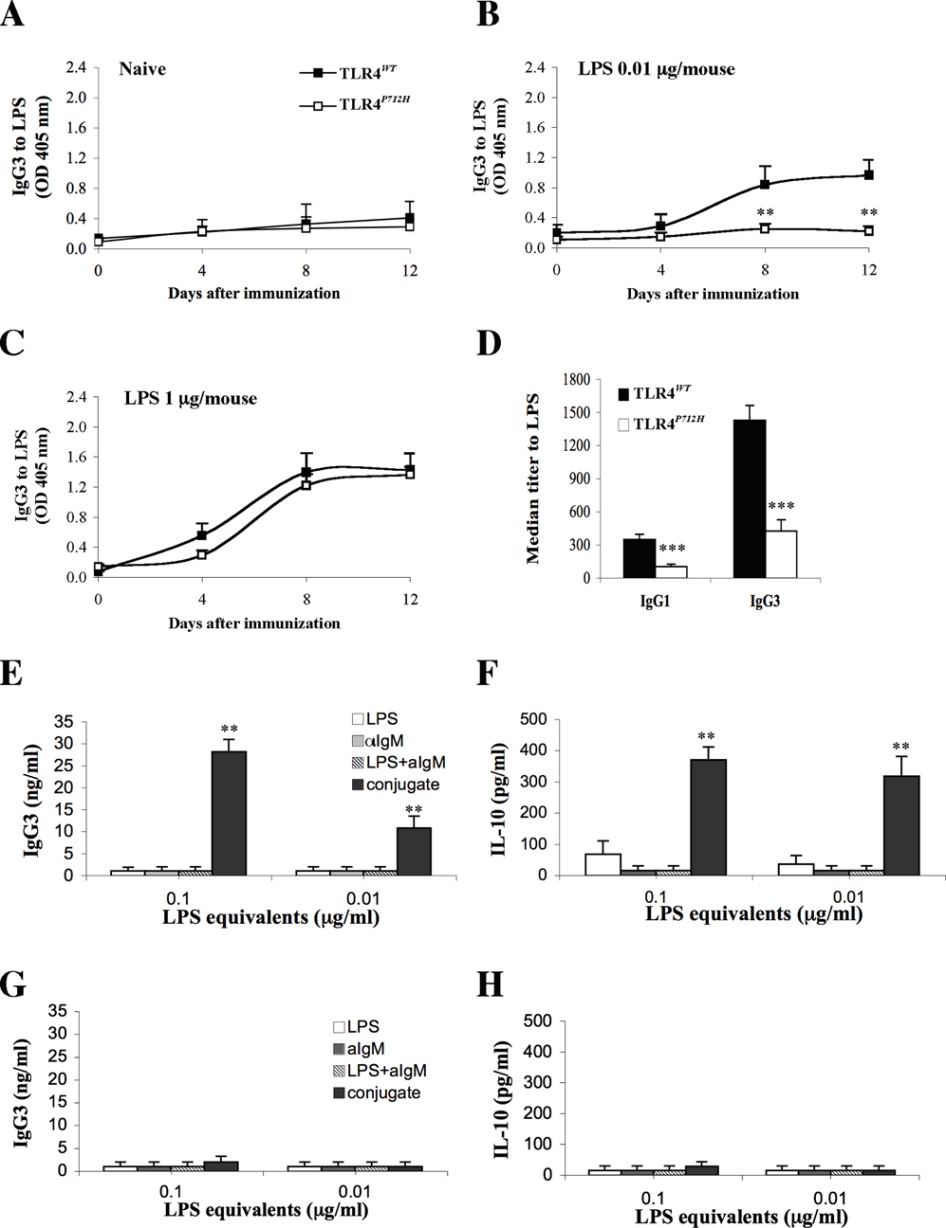
TLR4 and BCR synergize. A–C. TLR4*^WT^* and TLR4*^P712H^* mice were left untreated (A) or immunized with a single low (B, 0.01 µg/mouse) or high (C, 1 µg/mouse) dose of LPS in PBS and IgG3 antibodies to LPS were determined by ELISA. ** *P*<0.01 when compared to TLR4*^WT^* mice. D. The titer of antibodies to LPS was determined in the serum samples taken 12 days after immunization with 1 µg/mouse of LPS. Results represent the mean (±SD) of each experimental group (n = 5, *** *P*<0.001 when compared to TLR4*^WT^* mice). E and F. Purified B cells from TLR4*^WT^* mice were activated *in vitro* with LPS, αIgM (aIgM), LPS and αIgM (LPS+aIgM) or the [LPS-aIgM] conjugate (conjugate) for 48 hr, and the supernatants were assayed for the secretion of IgG3 (E) or IL-10 (F). ** *P*<0.01 when compared to cells treated with LPS, αIgM (aIgM) or LPS and αIgM (LPS+aIgM). Purified B cells from TLR4*^P712H^* mice were activated *in vitro* with LPS, αIgM (aIgM), LPS and αIgM (LPS+aIgM) or the [LPS-aIgM] conjugate (conjugate) for 48 hr, and the supernatants were assayed for the secretion of IgG3 (G) or IL-10 (H).

We tested whether functional TLR4 was also needed for the induction of IgG3 by deliberate immunization to LPS. We immunized 1-month old TLR4*^WT^* or TLR4*^P712H^* mice with LPS. At this age (**Figure 1C**) and throughout the experiment (**Figure 3A**), un-immunized mice of both strains were still negative for natural anti-LPS IgG antibodies. Groups of mice received a single high (1 µg) or low (0.01 µg) dose of *E. coli* LPS, or 1 µg of *P. gingivalis* LPS (pgLPS), or PBS, and we assayed the mouse sera for anti-LPS antibodies. Immunization with 0.01 µg of *E. coli* LPS sufficed to induce anti-LPS IgG3 in the TLR4*^WT^* mice, and to a lesser extent IgG1 (**Figures 3B and 3D**). In contrast, the 0.01 µg dose of LPS did not induce the TLR4*^P712H^* mice to produce anti-LPS IgG antibodies (**Figure 3B**). Both the TLR4*^WT^* and the TLR4*^P712H^* mice produced anti-LPS IgG (detected at a dilution of 1∶100) upon immunization with the 1 µg dose of LPS (**Figure 3C**). Nevertheless, serial dilutions of the sera demonstrated that the anti-LPS IgG3 titers were significantly higher in the TLR4*^WT^* mice (**Figure 3D**, p<0.05). The antibodies to *E. coli* LPS were specific; immunization with pgLPS did not induce significant levels of anti- *E. coli* LPS IgG antibodies in either strain, and mice immunized with *E. coli* LPS did not manifest cross-reactivity to pgLPS (data not shown). Moreover, no differences in LPS-specific IgM levels were detected between the two strains of mice upon immunization with the 0.01 µg or the 1 µg dose of LPS (data not shown). These results indicate that TLR4 signaling is critical not only for natural anti-LPS IgG3 antibodies but also for the induction of anti-LPS IgG3 antibodies in situations where antigen availability is the limiting factor. Higher doses of LPS can apparently generate IgG3 antibodies by other signaling pathways, although the antibody titer, often a critical factor in protection, is still controlled by the TLR4-generated signal.

### TLR4 and BCR synergize

LPS is composed of a lipid A moiety connected to chains of polysaccharide. Lipid A is responsible for the toxic effects of LPS and the B-cell mitogenic effect, however it is virtually non-immunogenic; antibodies to LPS bind to the polysaccharide chains [27]. The finding that TLR4 signaling is needed for the induction of IgG3 antibodies to LPS in intact mice suggested that the innate TLR4, activated by the lipid A component of LPS, and the adaptive BCR molecules that recognize polysaccharide LPS epitopes, might interact together in responding B cells during activation. To test this hypothesis, we studied the responses of purified B cells to LPS and/or BCR stimulation in vitro. We simulated BCR antigen-activation using mitogenic anti-IgM antibodies (αIgM) that cross-link the BCR. *In vitro*, αIgM does not induce isotype switching to IgG3 whereas LPS is a mitogenic signal that does induce switching to IgG3, but at relatively high concentrations (**Figure S2** and ([28]). IgG3 synthesis is known to require the autocrine secretion of IL-10 by activated B-cells [29]. Thus we could use αIgM and LPS to detect possible synergistic effects of the activation of BCR- and TLR4-dependent signaling pathways, respectively.

For *in vitro* experiments, we purified B cells from the spleens of TLR4*^WT^* or TLR4*^P712H^* mice, and then measured B-cell proliferation, secretion of IL-10 and production of IgG3 in response to the following agents: LPS alone, αIgM alone, mixtures of LPS and αIgM, and LPS conjugated to αIgM. The conjugate simulates the encounter of LPS by a B-cell bearing a BCR specific for the polysaccharide since the antigenic polysaccharide epitope and the lipidic TLR4 ligand are covalently bound in the LPS molecule. The purified population of B cells was free of T cells or macrophages (data not shown), and thus there was no T-cell help. Surprisingly, despite no differences in B-cell proliferation to these mitogenic agents (**Figure S3**), the LPS-αIgM conjugate triggered only the TLR4*^WT^* B cells to secrete significantly higher levels of IL-10 and IgG3 compared to the other stimuli (**Figures 3E and 3F**). The effect of the conjugate was mediated by TLR4: we did not detect IL-10 or IgG3 secretion from purified TLR4*^P712H^* B-cells stimulated by the conjugate (**Figures 3G and 3H**). Since conjugation of the two mitogens was required to induce the production of IgG3, it was likely that co-ligation of TLR4 and BCR was sufficient to activate the IgM to IgG3 switching mechanism in the absence of T cells.

### TLR4 and BCR associate on the B cell membrane

Costimulation of B cells through different co-receptors is known to modulate B cell activation [30]. The CD19-CD21 complex, for example, co-clusters with the BCR upon interaction with complement-tagged antigens resulting in increased BCR signaling [30], thus we studied the surface distribution of the BCR and TLR4 molecules by confocal microscopy. Membrane IgM and TLR4 molecules capped together on the surface of B-cells following *in vitro* activation with LPS; these caps also contain FITC-labeled LPS (**Figure S4A**). An additional TLR family member expressed on B cells, RP105, was not recruited to the LPS-induced caps (data not shown) [31]. Thus, B-cell activation by LPS triggers the co-localization of TLR4 and BCR molecules.

**Figure 4:**
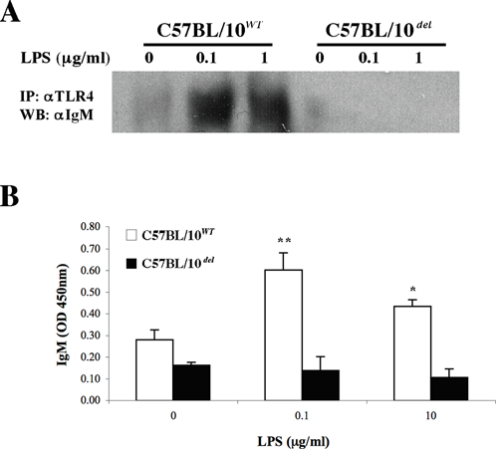
TLR4 and BCR interact in the membrane of LPS-activated B-cells. A. Purified B cells from TLR4*^WT^* or TLR4*^del^* mice were incubated for 1 hr with 0, 0.1 or 1 µg/ml of LPS, TLR4 was immunoprecipitated and bound proteins were separated by PAGE-SDS and analyzed by western blot using an IgM-specific antibody. D. Purified B cells from TLR4*^WT^* or TLR4*^del^* mice were incubated for 1 hr with 0, 0.1 or 1 µg/ml of LPS, TLR4 was immunoprecipitated with a specific biotinylated antibody and then captured on streptavidin-coated microplates and bound IgM was detected with a specific HRP-conjugated antibody. * *P*<0.05 and ** *P*<0.01 vs. control (no LPS).

To confirm these results, we performed immuno-precipitation and pull-down assays. IgM and TLR4 were co-precipitated from extracts prepared from wild-type LPS-treated B cells, but not from TLR4*^del^* B-cells (**Figure 4A** **and** **Figure S4B**). No co-precipitation was detected using extracts from LPS-treated TLR4*^P712H^* B cells, suggesting that physical association depends on functional signaling through TLR4 (data not shown). To confirm these results, we preincubated cell extracts from LPS activated (or control) B cells with biotinylated antibodies to TLR4, and then captured the biotinylated antibody-antigen complexes on streptavidin coated microtiter plates. The plates where washed and the BCR molecules pulled-down with TLR4 were quantified using an HRP-conjugated anti-mouse IgM detection antibody. TLR4/IgM complexes could be pulled down from TLR4*^WT^* B cells, but not from their TLR4*^del^* counterparts (**Figure 4B**). These results suggest that the TLR4 and IgM molecules are associated on the surface of LPS-activated B cells, either directly or through yet uncharacterized interaction partners.

### TLR4/BCR association does not require CD14 and MD-2 and is specific to TLR4

**Figure 5:**
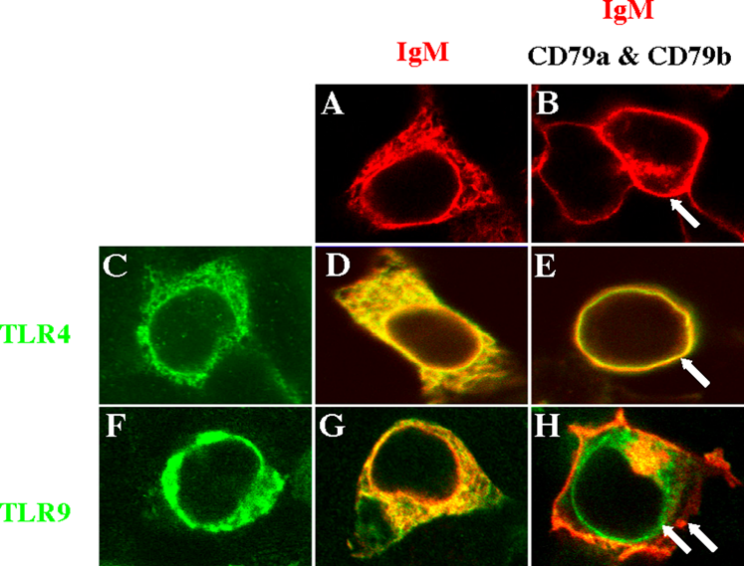
TLR4 and BCR are physically associated. HEK293 cells were transfected with BCR and HA-tagged TLR constructs, fixed, mounted, and examined by confocal microscopy. TLR staining (anti-HA followed by Cy3-conjugated anti- mouse IgG) was pseudo-colored green and IgM staining (with Cy5-conjugated anti-human IgM) was pseudo-colored red. Panels showing TLR/BCR co-transfection experiments (D–E and G–H) represent an overlay of the green and red channels. A and B. IgM HC and LC constructs (BCR, shown in A) are fully cytoplasmic (A) but they are shifted to the membrane upon transfection with CD79a and CD79b coding constructs (B, arrow). C–H. HA-tagged TLR4 (C–E) or TLR9 (F–H) were transfected alone (C and F) or co-transfected with the IgM HC and LC constructs (D and G) or HC/LC+CD79a/CD79b (E and H). HA-tagged TLR4 (C–E), but not HA-tagged TLR9 (F–H), is dragged to the membrane upon cotransfection with CD79a and CD79b coding constructs.

To characterize the components involved in the interaction between TLR4 and BCR, we used the non-lymphoid HEK293 cell line. Transfection of HEK293 cells with vectors coding for the IgM heavy and light chains (HC and LC, respectively) leads to the cytoplasmic localization of the newly made IgM (**Figure 5A**); the IgM is relocalized to the cell surface upon co-transfection with vectors coding for CD79a and CD79b (**Figure 5B**) [32]. Since HEK293 cells can also express functional TLR4 [33], we studied whether the interaction between TLR4 and the BCR might result in changes in the localization of these molecules. Transfection of HEK293 cells with vectors coding for TLR4 (**Figure 5C**) alone or together with IgM HC and LC (**Figure 5D**) led to the cytoplasmic expression of the newly synthesized proteins. The cytoplasmic localization of TLR4 in HEK293 cells has been reported previously and may be related to the absence of additional components of the LPS receptor complex in these cells (find ref). In any case, the cytoplasmic location of TLR4 when expressed alone or together with IgM HC and LC dramatically shifted when TLR4 was co-transfected with IgM HC and LC together with CD79a and CD79b. Co-transfection led to the translocation of both the newly synthesized IgM and TLR4 to the cell membrane (**Figure 5E**). Thus, the interaction evidenced by co-precipitation experiments shown in Figure 4 appears to be operational in HEK293 cells, allowing BCR molecules to drag the TLR4 to the cell surface. Since the transfected cells express TLR4 but not CD14 or MD-2 [33], these components of the LPS receptor complex do not appear to be involved in the TLR4 association with the BCR. Note however, that in HEK293 cells TLR4 physically associated with the BCR in the absence of LPS stimulation, probably as a result of the over-expression of the transfected molecules or because of differences in membrane microdomains of this cell line and primary B cells [34].

Since synergism between the BCR and TLR9 has also been described [7], [8], [35], [36], [37], we studied whether TLR9 could interact with the BCR in HEK293 cells. In contrast to TLR4, co-transfection of HA-tagged TLR9 with IgM HC/LC and CD79a/CD79b, led to the cytoplasmic expression of TLR9 and surface localization of the IgM (**Figures 5F–H**). This suggests that TLR9 does not associate physically with the BCR in this system. Since TLR9 is known to localize to intracellular compartments in all cells studied [38], overexpression in HEK293 cells with the BCR would not be expected to shift its expression to the cell surface. The fact that TLR9 expression remained intracellular validated the use of HEK293 transfectants in this study of TLR trafficking and localization. Nevertheless, in B cells the BCR is internalized following antigen cross-linking and direct or indirect interaction with intracellular TLR9 may underlie the synergistic signal [38], [39].

### The transmembrane domain of TLR4 mediates the interaction with the BCR

**Figure 6:**
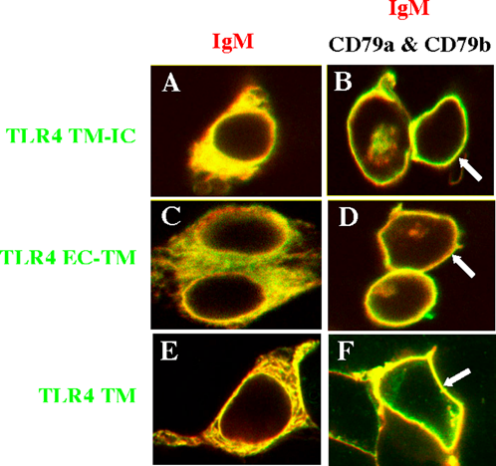
TLR4/BCR associate through the TLR4 transmembrane region. HEK293 cells were co-transfected with truncated or full length TLR4 constructs with BCR (*left panel*) or BCR+CD79a/CD79b (*right panel*). Cells were prepared as in Figure 5 and all panels represent the overlay of green (anti-HA staining) and red (anti-IgM staining) channels. The HA-tagged TLR4 TM-IC construct (A–B), TLR4 TM-EC construct (C–D), as well as the TLR4 TM region alone (E–F) each translocated to the cell surface with expression of surface IgM.

**Figure 7:**
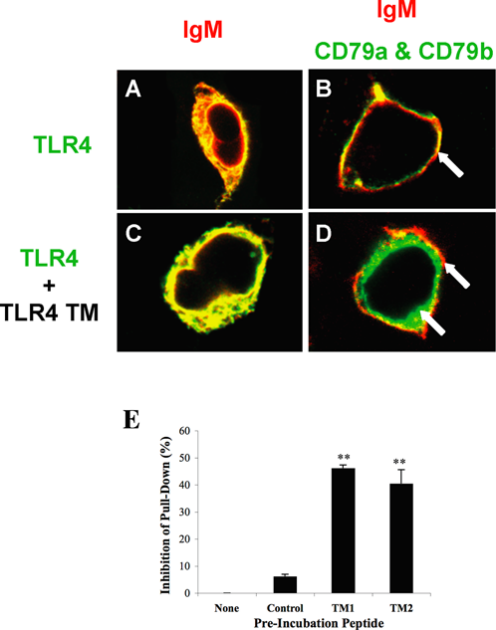
The TLR4 transmembrane region competitively inhibits the TLR4/BCR association. A–D. HEK293 cells were co-transfected with HA-tagged full length TLR4, along with the BCR (A and C) or the BCR and CD79a/CD79b constructs (B and D). In addition, cells in C and D were also cotransfected with a construct coding for the unlabeled TLR4 TM region. Cells were prepared as in Figure 5 and all panels represent the overlay of green (anti-HA staining) and red (anti-IgM staining) channels. The non-tagged TLR4 TM construct competed with the HA-TLR4 TM-IC, inhibiting its translocation to the cell membrane (compare B to D). E. Purified B cells from TLR4*^WT^* mice were pre-incubated for 2 hr with peptides TM1 or TM2 corresponding to the transmembrane region of TLR4 or with a control peptide, activated with 1 µg/ml of LPS for 1 hr, and TLR4 was immunoprecipitated with a specific biotinylated antibody and captured on streptavidin-coated microplates. Bound IgM was detected with a specific HRP-conjugated antibody as in figure 5C. ** *P*<0.01 vs. control-treated group.

To map the domain in the TLR4 molecule responsible for the TLR4/BCR interaction, we studied the ability of truncated TLR4 proteins to interact with IgM and relocalize to the membrane of HEK293 cells upon cotransfection with CD79a and CD79b. We used HA-tagged TLR deletion mutants containing the extracellular and transmembrane domains (named TLR4 EC-TM), intracellular and transmembrane domains (TLR4 TM-IC) or only the transmembrane domain (TLR4 TM). TLR4 TM-IC (**Figures 6A and B**), TLR4 EC-TM (**Figures 6C and D**) and TLR4 TM (**Figures 6E and 6F**) trafficked to the membrane when IgM HC and LC were co-expressed with CD79a and CD79b, suggesting that the TLR4-BCR interaction is mediated by the TLR4 transmembrane domain. To test this hypothesis, we made a construct coding for a non-HA tagged version of the 38 aa TLR4 transmembrane domain. Over-expression of this non-tagged TLR4 transmembrane domain competed with the HA-tagged full length TLR4 and inhibited its translocation to the cell membrane upon cotransfection with CD79a and CD79b (**Figure 7A–D**). Therefore, the TLR4/BCR interaction in HEK293 cells is mediated by the TLR4 transmembrane domain.

To analyze whether the TLR4/BCR interaction was also mediated by the TLR4 transmembrane domain in the membrane of LPS-activated B cells, we competed the pull-down of IgM and TLR4 molecules (**Figure 4B**) by preincubation with peptides coding for the TLR4 transmembrane domain (10 µM). Peptide TM1 corresponds to the 648–659 aa region of TLR4, while peptide TM2 corresponds to the 629–655 aa region of TLR4. As a control we used a peptide derived from HSP60 that shows a similar charge distribution (VLGGGCALLRCPALDSLTPANED). Preincubation with the TM1 or TM2 peptides, but not with a control peptide, led to a dose-dependent decrease in the IgM pulled down with biotinylated antibodies to TLR4 from LPS-activated cell extracts (**Figure 7E**). Thus, the TLR4 TM domain mediates the TLR4/BCR interaction on B-cells following exposure to LPS.

## Discussion

In this work we report that TLR4 synergizes with the BCR to control the induction of IgG3 antibodies to LPS. The control exerted by TLR4 in the induction of anti-LPS IgG is the result of synergism between TLR4 and BCR signaling pathways triggered with limiting amounts of LPS in the absence of T-cell help. This synergistic interaction manifested three characteristics: A) It was constrained by the specificity of the TLR involved; inactivating mutations in TLR4 did not decrease the induction of antibodies to a TLR2 ligand. B) It was shown to be MyD88-dependent; synergism was not observed in MyD88-deficient mice. C) It was mediated by interactions that depended on the transmembrane domain of TLR4 and the BCR. Taken together, our results show that synergism between TLR4 and the BCR lowers the threshold for IgG3 production by B cells expressing an LPS-reactive BCR. Note that the control exerted by TLR4 in the induction of anti-LPS IgG3 might operate at the level of the class-switch recombination (CSR) to IgG3 and/or the somatic hypermutation (SHM) of LPS-specific antibodies. Both CSR and SHM are mediated by the activation-induced cytidine deaminase (AID) [40], and AID expression levels have been shown to be regulated by TLR signaling [41], [42]. Although our data using in vitro activated purified B cells shows a direct effect of TLR4 on CSR (Figures 3E–H), the decreased levels of LPS-specific IgG3 observed in mice carrying a mutant TLR4 might reflect a decrease in both CSR and SHM.

Our results support a role for TLRs in controlling the antibody response to TLR-ligands under conditions where the antigen is present in limiting amounts. Accordingly, natural IgG3 (and to a lesser extent natural IgG1) antibodies to LPS could only be detected in animals harboring a functional TLR4/MyD88 pathway. TLR-dependency, however, was restricted to control of production of IgG3 antigen specific antibodies, since we detected no association between anti-LPS IgM levels and the presence of a functional TLR4/MyD88 pathway. It has been recently reported that MyD88-dependent signaling in B cells is required for the generation of IgM and IgG1 antibodies to the TLR5 ligand flagellin, while MyD88 signaling in non-B cells might be needed for the generation of specific IgG3 antibodies [12]. Flagellin, however, is a bacterial protein that in addition to its role as a TLR5 ligand [43] also provides T-cell epitopes and consequently T-cell help for the induction of an anti-flagellin B-cell response [12]; moreover, to study the induction of the anti-flagellin antibody response the authors complexed the immunogen to alum [12]. Nevertheless, the results of Pasare and Medzhitov [12] and our own work suggest that TLR signaling in B cells controls aspects of the B cell response to TLR ligands.

Synergistic signaling has also been shown for the BCR and TLR9, and the dual engagement of these receptors is implicated in the triggering of IgM to IgG switching events and activation of autoreactive B cells [7], [8], [9]. Co-signaling seems to depend on physical interaction, either direct or indirect, in the case of TLR4/BCR, but may be independent of physical association in the case of TLR9. Endogenous TLR9 is sequestered in intracellular compartments in both resting and activated B-cells [44], supporting the notion that synergism is generated downstream of receptor-ligand binding events. Recently, however, Chaturvedi et al demonstrated that the subcellular location of TLR9 is shifted to compartments containing the internalized BCR in B cells following cross-linking of the BCR on the surface by antigen [39]. Thus, TLR9 and BCR may associate in subcellular compartments although the fact that the TLR4/BCR association mapped to the TLR4 TM domain in our study, suggests that this interaction with the BCR, whether directly or through additional partners, may be unique to TLR4. The TLR4 TM domain is highly conserved across species (80% homology between mouse and human), but there is little sequence similarity between TM domains of different TLR family members. The structural motifs that mediate the BCR/TLR4 interaction are therefore likely to be absent from other TLR TM domains. Thus, the synergism of the BCR with TLR4 seems to operate following rules different from those governing the crosstalk with other TLRs.

Conversely, intermolecular associations seem to play a central role in the regulation of TLR4-dependent signal transduction and the response to LPS. It has been recently been reported that TLR4 interacts in DC with RP105, and this interaction results in the modulation of the response to LPS [45]. Although it has still to be proven that this mechanism is operative in B cells, it suggests that the partition of TLR4 between different interacting partners might play a role in modulating the B-cell response to LPS.

The mitogenic properties of LPS and its T-cell independence were described long before TLRs were discovered [46]. Our results provide a new molecular understanding for how IgG antibodies to LPS arise. The interaction of TLR4 with an LPS-reactive BCR results in a synergistic signal that induces IgG3 and IL-10 production, in the absence of T-cell help. Although the synergistic signal may be achieved by surface co-ligation of the BCR and TLR4 in the absence of an association of the receptors, the intimate relationship we describe enhances the sensitivity to the signal, allowing IgG3 antibodies to arise in a setting of very low circulating levels of LPS. Sub-mitogenic levels of LPS might also impact upon other functions of B-cells through BCR-TLR4 synergy, such as antigen uptake and presentation to T-cells and antibody somatic mutation [12], [47].

The control of the induction of specific IgG3 antibodies triggered by limiting amounts of LPS might be clinically important. IgG3 antibodies contribute to the control of viral and bacterial infections through opsonization, complement fixation, and FcR mediated activation of neutrophils [48]. IgG3 anti-LPS antibodies have also been described to sequester LPS in the circulation thereby preventing or reducing its toxicity [49]. Although other mechanisms might also be involved, a lack of TLR4-BCR synergism may contribute to the increased susceptibility to *E. coli* or *S. typhimurium* infection in TLR4 deficient mice [50], [51], and to the increased risk of gram-negative infections in humans carrying inactivating TLR4 polymorphisms [52]. Thus, the synergistic signaling between the BCR and innate receptors may be a general property of B-cell physiology important for the generation of microbe-specific antibodies in the early stages of an immune response when antigen specific T cell help is not yet available. These synergistic interactions are potential targets for the design of new vaccines aimed at exploiting the innate-adaptive cross-talk to provide fast and effective protective immunity against pathogens.

## Materials and Methods

### Mice

NOD/LtJ, C3HeB/FeJ and C3H/HeJ mice (Jackson Laboratories, Maine, USA) were bred and maintained in the spf animal facility of the Weizmann Institute of Science. C57BL/10 (herein referred to as TLR4^++^) and C57BL/10ScNJ-Tlr4*^lps-del^* (herein referred to as TLR4*^del^*) were from The Jackson Laboratory and were housed in the spf of Harvard Medical School. All experimental protocols were approved by the Institutional Animal Care and Use Committees of the respective institutions.

### Sera

Blood was taken from the lateral tail vein and sera was stored at −20°C.

### ELISA assay

Antibodies to gel purified LPS of *E. coli* 055:B5 (Sigma, MO, USA, product #L2637) were measured by ELISA. Plates (Maxisorb Nunc, Denmark) coated overnight with 50 ng/well of LPS were blocked with 1% BSA for 1 hr at 37°C. Serum samples diluted 1/100, unless otherwise stated, were added to the plates and incubated for 2 hr at 37°C. For the measurement of IgG reactivity, serum samples were pre-treated with 0.05 M β-mercaptoethanol to rule out masking by IgM [22]. Bound antibodies were detected using anti-mouse IgG or IgM (Jackson ImmunoResearch Labs. Inc.,West Grove, Pennsylvania, USA) or isotype-specific secondary antibodies (Southern Biotechnology Associates Inc, Birmingham, AL, USA) conjugated to alkaline phosphatase, and Sigma's substrate for alkaline phosphatase.

### C3H/HeJ X NOD/Ltj cross

F1 progeny of NOD female mice crossed with C3H/HeJ male mice were intercrossed and F2 tail vein DNA samples were screened to identify mice homozygous for the CCT→CAT mutation found in C3H/HeJ mice. A 400 bp DNA fragment surrounding the ScrF1 restriction site (200 bp on each side) located 25 bp downstream of the point mutation was amplified using the upstream primer ACCTGATACTTATTGCTGGCT and the reverse primer GCTAAGAAGGCGATACAATTC. PCR products were cleaned, digested with ScrF1 (New England Biolabs, MA, USA), and separated on 3% agarose gels (Cambrex Bio Science Rockland Inc,, Rockland, ME, USA). The CCT→CAT mutation causes a loss of a ScrF1 endonuclease site and therefore DNA samples homozygous for the mutation are digested at only one site and yield two products of equal length (200 bp). Homozygous wt PCR products are cut at two sites and yield two visible bands of equal intensity, one 200 bp in length and the other 175 bp. It follows that 75% of the products from DNA of heterozygote samples is 200 bp in length and 25% is 175 bp. Homozygous mutant mice were backcrossed for three generations onto the NOD/Ltj background.

### Conjugation of LPS to anti-IgM

LPS was conjugated to Goat anti-mouse IgM (Jackson ImmunoResearch, West Grove, Pennsylvania, USA) using the heterobifunctional cross-linking reagent EMCH (Pierce, Rockford, IL, USA). The anti-IgM was thiolated using 2-Iminotiholane HCl at a 5-fold molar excess of thiolane to IgG. The reaction was purified by gel filtration with a Sephadex G-25 column (Pierce). Thiolated IgG was reacted with a 10-fold molar excess of EMCH for 2 h at RT. In parallel, LPS was oxidized with 10 mM sodium periodate (Sigma, Rehovot, Israel) for 30 min at RT. Oxidized LPS was added to the IgG-EMCH reaction at a 10-fold molar excess of hydrazide to LPS and reacted for 2 h at RT. The product was purified by size exclusion chromatography and LPS content of the conjugate was determined using the kinetic-turbidimetric LAL test method compared to a standard control (Biological Industries, Kibbutz Beit Haemek, Israel).

### B cell purification and activation

Splenic B cells were purified from 2 month old mice using the MACS B Cell Isolation Kit (Miltenyi Biotec GmbH, Bergisch Gladbach, Germany) according to the manufacturer's directions. The purity of the purified B cell fraction was >97% as determined by FACS analysis (data not shown).

Purified B cells were cultured in quadriplicates (2×10^5^/well) in round-bottom plates (Nunclon, Nunc, Denmark) for 72 hr at 37°C in a humidified atmosphere with 7.5% CO_2_, in the presence of different concentrations of αIgM, LPS or the [αIgM-LPS] conjugate. Stimulation medium was composed of Dulbecco's modified Eagle's medium (Gibco, Paisley, UK) supplemented with 2-mercaptoethanol (5 10^−5^ M, Sigma, Rehovot, Israel), L-glutamine (2 mM, Biological Industries, Kibbutz Beit Haemek, Israel), sodium pyruvate (1 mM, Sigma, Rehovot, Israel), penicilin and streptomycin (100 u/ml and 100 µg/ml, respectively; Biological Industries, Kibbutz Beit Haemek, Israel), non-essential amino acids (1% v/v, Bio Lab, Jerusalem, Israel), and 5% fetal calf serum (Grand Island Biological Company, Grand Island, NY, USA). [^3^H]thymidine (0.5 µCi of 5 mCi/mmol; Amersham, England) was added to the cultures for the last 18 hr of incubation. Thereafter, supernatants were collected, cells were harvested and the cpm were counted. The results are expressed as the stimulation index, the mean cpm of cultures incubated with stimulant divided by the mean cpm of cultures incubated in the absence of stimulant.

### IgG3 and IL-10 secretion

B-cell culture supernatants were analysed for IgG3 and IL-10 content by ELISA using an appropriate pair of capture and detecting monoclonal antibodies (IL-10: Pharmingen, San Diego, USA; IgG3: Southern Biotechnology Associates Inc, Birmingham, AL, USA) according to the manufacturer's instructions. The results are expressed as pg/ml, based on a calibration curve constructed using known amounts of recombinant IgG3 (Southern Biotechnology Associates Inc) or IL-10 (Pharmingen).

### Immunoprecipitation and western blotting

Purified B cells (2×10^6^) were lysed for 15 min on ice in 0.1 ml lysis buffer containing 1% digitonin (Sigma, Rehovot, Israel). Insoluble material was removed by centrifugation at 10.000 g for 10 min at 4 C. After pre-clearing of the lysate with Protein G-plus Agarose beads (Santa Cruz Biotechnology Inc., Santa Cruz, California, USA) the IgM molecules were precipitated using biotinylated anti-IgM antibodies (Jackson ImmunoResearch Inc.) or biotinylated anti-TLR4 (eBioscience, San Diego California, USA) followed by ON incubation with streptavidin agarose beads (Sigma). The beads were washed with lysis buffer, boiled, and the protein supernatant run in a 10–15% SDS-PAGE and analyzed by western blot using HRP conjugated antibody to IgM (Jackson ImmunoResearch Labs. Inc.) or TLR4 (eBioscience, USA) and the Western Blotting Luminol Reagent (Santa Cruz Biotechnology Inc.). Alternatively, lysates were precipitated with biotinylated anti-TLR4 and incubated on streptavidin coated plates ON. HRP-conjugated anti-IgM was used to reveal bound complexes using and OD was recorded at OD_450_. We used the following unrelated peptide as a control: VLGGGCALLRCPALDSLTPANED.

### Confocal microscopy

Purified B-cells were incubated overnight with LPS (20 µg/ml) or left untreated, fixed with 4% para-formaldehyde for 15 min on ice and washed with PBS. The cells were divided into aliquots containing 50,000 cells per 100 µl and incubated with biotinylated Fab anti-IgM (Jackson ImmunoResearch Inc.), antibodies to TLR4 or RP105 (Santa Cruz Biotechnology Inc.), or FITC-conjugated *E. coli* LPS (Sigma). The cells were washed, incubated with streptavidin conjugated to Cy3 (Jackson ImmunoResearch Inc.) or Cy5-conjugated anti-rat antibodies (Jackson ImmunoResearch Inc.) and adhered onto Poly-lysine microscope slides (BDH Laboratory Supplies, Poole,UK). Immunofluorescence was viewed and analyzed using a Bio-Rad confocal microscope (Bio-Rad, Richmond, CA).

### Plasmids and cloning

pDUO-hMD2/CD14 and pUNO-hTLR4HA (encoding HA-tagged TLR4) were purchased from Invivogen (San Diego, CA). pDISPLAY encoding HA-tagged TLR9 was a gift from Dr. David Segal (National Cancer Institute, National Institutes of Health, Bethesda, MD). IgM mu and kappa chains, CD79a and CD79b were cloned using RT-PCR and inserted in-frame into pORF (Invivogen). RNA for mu and kappa chains was extracted from SP2/0 cells expressing anti-TNP IgM [53], a gift from Dr. Mark Shulman (University of Toronto, Toronto, Canada). RNA for CD79a and CD79b was extracted from the Burkitt lymphoma Raji cell line, a gift from Dr. Eithan Galun (Hadassah Medical Center, Jerusalem, Israel).

### Transfection and cell staining

HEK293 cells were a gift from Dr. Eitan Galun (Hadassah Medical Center). For stable expression of HA-TLR4, cells were transfected in 6-well plates using TransIT LT1 transfection reagent according to the manufacturer's protocol (Mirus, Madison, WI) and then selected with Blasticidin S (10 µg/ml). For transient transfection cells were plated on coverslips overnight followed the next day by transient transfection of the desired plasmids using TransIT LT1 transfection reagent. The next day cells were fixed using 4% PFA in PBS, washed, and incubated with Cy5-conjugated anti-human IgM (Jackson immunoresearch laboratories Inc.), and monoclonal anti-HA (Covance Research Products, Berkeley, CA, USA), followed by secondary antibody Cy3-conjugated anti- mouse IgG (Jackson immunoresearch laboratories Inc.). Coverslips were mounted in 70% glycerol and visualized and analyzed using an Olympus FluoView 300 laser scanning confocal microscope.

### Statistical significance

The Prism 4.0a program (GraphPad Software, La Jolla, CA, USA) was used for statistical analysis. Student's *t*-test, and one-way and two-way ANOVA tests were carried out to assay significant differences between the different experimental groups.

## Supporting Information

Table S1 (0.15 MB DOC)

Figure S1 (0.08 MB DOC)

Figure S2 (0.35 MB DOC)

Figure S3 (0.36 MB DOC)

Figure S4 (0.30 MB DOC)

## References

[1] Stavnezer J (2000). Molecular processes that regulate class switching.. Curr Top Microbiol Immunol.

[2] Chaudhuri J, Alt FW (2004). Class-switch recombination: interplay of transcription, DNA deamination and DNA repair.. Nat Rev Immunol.

[3] Akira S (2006). TLR signaling.. Curr Top Microbiol Immunol.

[4] Schnare M, Barton GM, Holt AC, Takeda K, Akira S (2001). Toll-like receptors control activation of adaptive immune responses.. Nat Immunol.

[5] Caramalho I, Lopes-Caravalho T, Ostler D, Zelenay S, Haury M (2003). Regulatory T cells selectively express toll-like receptors and are activated by lipopolysaccharide.. J Exp Med.

[6] Bernasconi NL, Traggiai E, Lanzavecchia A (2002). Maintenance of serological memory by polyclonal activation of human memory B cells.. Science.

[7] Christensen SR, Kashgarian M, Alexopoulou L, Flavell RA, Akira S (2005). Toll-like receptor 9 controls anti-DNA autoantibody production in murine lupus.. J Exp Med.

[8] Ehlers M, Fukuyama H, McGaha TL, Aderem A, Ravetch JV (2006). TLR9/MyD88 signaling is required for class switching to pathogenic IgG2a and 2b autoantibodies in SLE.. J Exp Med.

[9] He B, Qiao X, Cerutti A (2004). CpG DNA induces IgG class switch DNA recombination by activating human B cells through an innate pathway that requires TLR9 and cooperates with IL-10.. J Immunol.

[10] Gavin AL, Hoebe K, Duong B, Ota T, Martin C (2006). Adjuvant-enhanced antibody responses in the absence of toll-like receptor signaling.. Science.

[11] Nemazee D, Gavin A, Hoebe K, Beutler B (2006). Immunology: Toll-like receptors and antibody responses.. Nature.

[12] Pasare C, Medzhitov R (2005). Control of B-cell responses by Toll-like receptors.. Nature.

[13] Alexopoulou L, Thomas V, Schnare M, Lobet Y, Anguita J (2002). Hyporesponsiveness to vaccination with Borrelia burgdorferi OspA in humans and in TLR1- and TLR2-deficient mice.. Nat Med.

[14] Akira S, Takeda K (2004). Toll-like receptor signalling.. Nat Rev Immunol.

[15] Coutinho A, Kazatchkine MD, Avrameas S (1995). Natural autoantibodies.. Curr Opin Immunol.

[16] Cohen IR, Norins LC (1966). Natural human antibodies to gram-negative bacteria: immunoglobulins G, A, and M.. Science.

[17] Macpherson AJ, Gatto D, Sainsbury E, Harriman GR, Hengartner H (2000). A primitive T cell-independent mechanism of intestinal mucosal IgA responses to commensal bacteria.. Science.

[18] Ochsenbein AF, Fehr T, Lutz C, Suter M, Brombacher F (1999). Control of early viral and bacterial distribution and disease by natural antibodies.. Science.

[19] Ochsenbein AF, Zinkernagel RM (2000). Natural antibodies and complement link innate and acquired immunity.. Immunol Today.

[20] Hoshino K, Takeuchi O, Kawai T, Sanjo H, Ogawa T (1999). Cutting edge: Toll-like receptor 4 (TLR4)-deficient mice are hyporesponsive to lipopolysaccharide: evidence for TLR4 as the Lps gene product.. J Immunol.

[21] Qureshi ST, Lariviere L, Leveque G, Clermont S, Moore KJ (1999). Endotoxin-tolerant mice have mutations in Toll-like receptor 4 (Tlr4).. J Exp Med.

[22] Scott DW, Gershon RK (1970). Determination of total and merecaptothanol-resistant antibody in the same serum sample.. Clin Exp Immunol.

[23] Durham JA, Antone SM, Cunningham MW, Confer AW (1988). Monoclonal antibodies to Pasteurella haemolytica serotype 1 lipopolysaccharide: demonstration of antigenic similarities among several serotypes.. J Clin Microbiol.

[24] Snapper CM, Marcu KB, Zelazowski P (1997). The immunoglobulin class switch: beyond “accessibility”.. Immunity.

[25] Quintana FJ, Cohen IR (2001). Autoantibody patterns in diabetes-prone NOD mice and in standard C57BL/6 mice.. J Autoimmun.

[26] Krieg AM (2002). CpG motifs in bacterial DNA and their immune effects.. Annu Rev Immunol.

[27] Miner KM, Manyak CL, Williams E, Jackson J, Jewell M (1986). Characterization of murine monoclonal antibodies to Escherichia coli J5.. Infect Immun.

[28] Ulevitch RJ, Tobias PS (1995). Receptor-dependent mechanisms of cell stimulation by bacterial endotoxin.. Annu Rev Immunol.

[29] Briere F, Servet-Delprat C, Bridon JM, Saint-Remy JM, Banchereau J (1994). Human interleukin 10 induces naive surface immunoglobulin D+ (sIgD+) B cells to secrete IgG1 and IgG3.. J Exp Med.

[30] Rickert RC (2005). Regulation of B lymphocyte activation by complement C3 and the B cell coreceptor complex.. Curr Opin Immunol.

[31] Ogata H, Su I, Miyake K, Nagai Y, Akashi S (2000). The toll-like receptor protein RP105 regulates lipopolysaccharide signaling in B cells.. J Exp Med.

[32] Fang T, Smith BP, Roman CA (2001). Conventional and surrogate light chains differentially regulate Ig mu and Dmu heavy chain maturation and surface expression.. J Immunol.

[33] Smith MF, Mitchell A, Li G, Ding S, Fitzmaurice AM (2003). Toll-like receptor (TLR) 2 and TLR5, but not TLR4, are required for Helicobacter pylori-induced NF-kappa B activation and chemokine expression by epithelial cells.. J Biol Chem.

[34] Pierce SK (2002). Lipid rafts and B-cell activation.. Nat Rev Immunol.

[35] Leadbetter EA, Rifkin IR, Hohlbaum AM, Beaudette BC, Shlomchik MJ (2002). Chromatin-IgG complexes activate B cells by dual engagement of IgM and Toll-like receptors.. Nature.

[36] Rifkin IR, Marshak-Rothstein A (2003). T-bet: the Toll-bridge to class-switch recombination?. Nat Immunol.

[37] Viglianti GA, Lau CM, Hanley TM, Miko BA, Shlomchik MJ (2003). Activation of autoreactive B cells by CpG dsDNA.. Immunity.

[38] Latz E, Schoenemeyer A, Visintin A, Fitzgerald KA, Monks BG (2004). TLR9 signals after translocating from the ER to CpG DNA in the lysosome.. Nat Immunol.

[39] Chaturvedi A, Dorward D, Pierce SK (2008). The B cell receptor governs the subcellular location of Toll-like receptor 9 leading to hyperresponses to DNA-containing antigens.. Immunity.

[40] Honjo T, Muramatsu M, Fagarasan S (2004). AID: how does it aid antibody diversity?. Immunity.

[41] Han JH, Akira S, Calame K, Beutler B, Selsing E (2007). Class switch recombination and somatic hypermutation in early mouse B cells are mediated by B cell and Toll-like receptors.. Immunity.

[42] Ueda Y, Liao D, Yang K, Patel A, Kelsoe G (2007). T-independent activation-induced cytidine deaminase expression, class-switch recombination, and antibody production by immature/transitional 1 B cells.. J Immunol.

[43] Hayashi F, Smith KD, Ozinsky A, Hawn TR, Yi EC (2001). The innate immune response to bacterial flagellin is mediated by Toll-like receptor 5.. Nature.

[44] Leifer CA, Kennedy MN, Mazzoni A, Lee C, Kruhlak MJ (2004). TLR9 is localized in the endoplasmic reticulum prior to stimulation.. J Immunol.

[45] Divanovic S, Trompette A, Atabani SF, Madan R, Golenbock DT (2005). Negative regulation of Toll-like receptor 4 signaling by the Toll-like receptor homolog RP105.. Nat Immunol.

[46] Spellman JM, Reed ND (1979). Immune and mitogenic responses by BALB/c, C3H/HeJ, and nude mice to Brucella abortus bacterin and lipopolysaccharide.. Infect Immun.

[47] Kinoshita K, Honjo T (2001). Linking class-switch recombination with somatic hypermutation.. Nat Rev Mol Cell Biol.

[48] Abbas AKLA, Pober JS (2000). Cellular and Molecular Immunology.

[49] Gianotti L, Braga M, Vaiani R, Almondo F, Di Carlo V (1996). Experimental gut-derived endotoxaemia and bacteraemia are reduced by systemic administration of monoclonal anti-LPS antibodies.. Burns.

[50] Hopkins WJ, Elkahwaji JE, Heisey DM, Ott CJ (2003). Inheritance of susceptibility to induced Escherichia coli bladder and kidney infections in female C3H/HeJ mice.. J Infect Dis.

[51] Hopkins WJ, Gendron-Fitzpatrick A, Balish E, Uehling DT (1998). Time course and host responses to Escherichia coli urinary tract infection in genetically distinct mouse strains.. Infect Immun.

[52] Agnese DM, Calvano JE, Hahm SJ, Coyle SM, Corbett SA (2002). Human toll-like receptor 4 mutations but not CD14 polymorphisms are associated with an increased risk of gram-negative infections.. J Infect Dis.

[53] Boulianne GL, Hozumi N, Shulman MJ (1984). Production of functional chimaeric mouse/human antibody.. Nature.

