# Supplementary material for: Induction of IgG3 to LPS via Toll-Like Receptor 4 Co-Stimulation
Source: PLoS One. 2008 Oct 23;3(10):e3509. doi: 10.1371/journal.pone.0003509 (PMC2566810; doi:10.1371/journal.pone.0003509)
Supplement: Table S1 — (0.15 MB DOC) [file pone.0003509.s001.doc]

**Table S1: Antigens used to probe the repertoire of natural antibodies.** The antigens are classified in different groups according to their cellular localization, tissue distribution or function.

| Group | Function / Structure | Antigen | Sigma  Catalogue |
| --- | --- | --- | --- |
| Cellular | Cytoskeleton | Actin | A3653 |
| Structure |  | Tubulin | T4925 |
|  |  | Myosin | M6643 |
|  |  | Tropomyosin | T4770 |
|  |  | Vimentin | V4383 |
|  | Extracellular | Fibronectin | F0895 |
|  | Matrix | Collagen I | C7774 |
|  |  | Collagen II | C7806 |
|  |  | Collagen III | C4407 |
|  |  | Collagen IV | C7521 |
|  |  | Collagen V | C3657 |
|  |  | Heparin | H2149 |
|  |  | Laminin | L6274 |
|  |  | Collagenase | C9891 |
| Cellular | Phospholipids | Cardiolipin | C5646 |
| Membranes |  | Glucocerebroside | G9884 |
|  |  | Phosphatidylethanolamine | P9137 |
|  |  | Cholesterol | C1145 |
| Cellular | Glucose | Enolase | E0379 |
| Metabolism |  | Aldolase | A8811 |
|  |  | Acid Phosphatase | P1774 |
|  | Apoptosis | Annexin 33 kDa. | A9460 |
|  |  | Annexin 67 kDa. | A2824 |
|  |  | Cytochrome P450C | C3131 |
|  | Monooxigenases | Catalase | C9322 |
|  |  | Peroxidase | P6782 |
|  |  | Tyrosinase | T 7755 |
|  | Others | Ribonuclease | R4875 |
| Nucleus | Protein | Histone II A | H 9250 |
|  | DNA | ssDNA | D1501 |
| Plasma Proteins | Carriers | Transferrin | T4132 |
|  |  | Fetuin | F2379 |
|  |  | Human Serum Albumin | A8763 |
|  |  | Rat Serum Albumin | A4538 |
|  |  | Ovoalbumin | A5378 |
|  | Coagulation | Factor II | F5132 |
|  |  | Factor VII | F6509 |
|  |  | Fibrin | F5386 |
|  |  | Fibrinogen | F4883 |
|  | Complement | C 1 | C2660 |
|  |  | C 1 q | C0660 |
| Immune System | Cytokines | Interleukin 2 | I2644 |
|  |  | Interleukin 10 | I9276 |
|  |  | Interleukin 4 | I4269 |
|  | Antibodies | Human IgG | I8640 |
|  |  | Human IgM | I8260 |
|  | TCR peptides | Pc9 | (a) |
|  |  | Pn4 | (b) |
| Tissue Antigens | Heat Shock | HSP60 | (c) |
|  | Proteins | p277 | (d) |
|  |  | HSP70 | (e) |
|  | Islet Antigens | GAD | G2126 |
|  |  | Insulin | I0259 |
|  | CNS | Human MOG | (f) |
|  |  | Human MOG p94-116 | (f) |
|  |  | Murine MOG | (f) |
|  |  | Rat MOG p35-55 | (f) |
|  |  | MBP | (g) |
|  |  | Brain Extract | B1877 |
|  | Muscle & | AchR | (h) |
|  | Skeleton | Myoglobulin | M6036 |
|  |  | Cartilage Extract | C5210 |
|  | Thyroid | Thyroglobulin | T1001 |
|  | Blood Cells & | Hemoglobin A | H0267 |
|  | Platelets | Spectrin | S3644 |
| Pathogens | Proteins | TB PPD | (i) |
|  | & Peptides | HSP65 | (j) |
|  |  | pEC27 | (k) |
|  |  | pMt278 | (l) |
|  |  | GST | (c) |
|  |  | KLH | (m) |
|  |  | Pepstatin | P5318 |
|  |  | Pr13 | (n) |
|  |  | *E. coli* LPS | L2637 |
| Synthetic | Poly | Poly Arginine | P3892 |
| Polymers | Amino | Poly Lysine | P4408 |
|  | Acids | Poly Aspartic | P6762 |
|  |  | Poly Glutamic | P4636 |
|  | Oligonucleotides | Poly A | (o) |
|  |  | Poly T | (p) |
|  |  | Poly C | (q) |
|  |  | Poly G | (r) |
|  |  | Poly ATA | (s) |
|  |  | Poly TAT | (t) |
|  |  | CpG-oligo | (u) |
|  |  | GpC-olilgo | (v) |

The name, number, catalogue number of Sigma (Rehovot, Israel) and source of each of the antigens used are indicated.

1. pC9: ASSLGGNQDTQY.
2. pN4: ASSLWTNQDTQY.
3. Purified as described (34).

(d) p277: VLGGGVALLRVIPALDSLTPANED.

(e) Recombinant human HSP70 was purchased from StressGen (San Diego, California, USA), catalogue number SPP-755.

(f) Kindly provided by Prof. Avraham Ben Nun, The Weizmann Institute, Israel. Human MOG p94-116: GGFTCFFRDHSYQEEAAMELKVE, rat MOG p35-55: MEVGWYRSPFSRVVHLYRNGK

(g) Kindly provided by Dr. Felix Mor, The Weizmann Institute, Israel.

(h) Kindly provided by Prof. Sara Fuchs, The Weizmann Institute, Israel.

(i) Produced at the Statens Seruminstitut, Copenhagen, Denmark.

(j) Kindly provided by Prof. Ruurd van der Zee, University of Utrecht, The Netherlands.

(k) pEC27: KKARVEDALHATRAAVEEGV.

(l) pMt278: EGDEATGANIVKVALEA.

(m) Purchased from Pierce (Oud Beijerland, The Netherlands), catalogue number 77153.

(n) pR13: EEEDDDMGFGLFD.

(o) Poly A: A_20_.

(p) Poly T: T_20_.

(q) Poly C: C_20_.

(r) Poly G: G_20_.

(s) Poly ATA: AT_18_A.

(t) Poly TAT: TA_18_T.

(u) TCCATAACGTTGCAAACGTTCTG.

(v) TCCATAAGCTTGCAAAGCTTCTG.
