# Supplementary material for: Induction of IgG3 to LPS via Toll-Like Receptor 4 Co-Stimulation
Source: PLoS One. 2008 Oct 23;3(10):e3509. doi: 10.1371/journal.pone.0003509 (PMC2566810; doi:10.1371/journal.pone.0003509)
Supplement: Figure S1 — (0.08 MB DOC) [file pone.0003509.s002.doc]

**Figure S1: Screening of NOD*^P172H^* mice.**

Mouse tail vein DNA samples were screened for the CCT→CAT mutation (first lane, 25bp marker – remaining lanes represent individual mice). Samples demonstrating a single band at 200bp following ScrF1 digestion are homozygous for CCT→CAT.


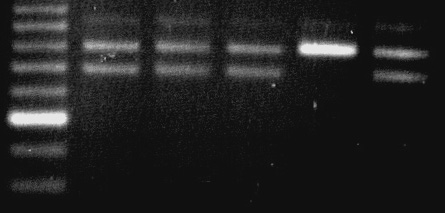


200bp

175bp

**NOD*^P172H^***

**NOD*^WT^* NOD*^WT^*  NOD*^WT^***
