# Supplementary material for: Induction of IgG3 to LPS via Toll-Like Receptor 4 Co-Stimulation
Source: PLoS One. 2008 Oct 23;3(10):e3509. doi: 10.1371/journal.pone.0003509 (PMC2566810; doi:10.1371/journal.pone.0003509)
Supplement: Figure S2 — (0.35 MB DOC) [file pone.0003509.s003.doc]

**Figure S2: IgG3 secretion upon stimulation with αIgM.**

Purified B cells from TLR4*^WT^* or TLR4*^P712H^* mice were activated *in vitro* with αIgM for 48 hr and the supernatants were assayed for the secretion of IgG3.

TLR4*^WT^*

TLR4*^P712H^*
