# Supplementary material for: Induction of IgG3 to LPS via Toll-Like Receptor 4 Co-Stimulation
Source: PLoS One. 2008 Oct 23;3(10):e3509. doi: 10.1371/journal.pone.0003509 (PMC2566810; doi:10.1371/journal.pone.0003509)
Supplement: Figure S3 — (0.36 MB DOC) [file pone.0003509.s004.doc]

**Figure S3: B cell activation with LPS and aIgM conjugate or wit LPS and aIgM trigger similar levels proliferation.**

Purified B cells from WT (A) or TLR4***^P712H^*** (B) mice were activated *in vitro* with LPS, αIgM (aIgM), LPS and αIgM (LPS+aIgM) or the [LPS-aIgM] conjugate (conjugate) for 48 hr and assayed for proliferation.

**A**

**B**
