# Supplementary material for: Induction of IgG3 to LPS via Toll-Like Receptor 4 Co-Stimulation
Source: PLoS One. 2008 Oct 23;3(10):e3509. doi: 10.1371/journal.pone.0003509 (PMC2566810; doi:10.1371/journal.pone.0003509)
Supplement: Figure S4 — (0.30 MB DOC) [file pone.0003509.s005.doc]

**Figure S4: TLR4 and BCR are colocalized on the cell membrane of activated B cells.** A. TLR4 and BCR are colocalized on the cell membrane of activated B cells. Purified B cells from TLR4***^WT^*** mice were incubated for 1 hr with or without LPS. The distribution of TLR4, IgM and LPS on the membrane was detected with TLR4- or IgM-specific antibodies followed by Cy5 or Cy3 secondary antibodies, or with FITC-labeled LPS, and the samples were visualized by confocal microscopy. B. Purified B cells from TLR4***^WT^*** mice were incubated for 1 hr with or without 1 mg/ml of LPS. Cell lysates were immunoprecipitated using control antibodies or antibodies to TLR4 or IgM and bound proteins were separated by PAGE-SDS and analyzed by western blot using an IgM-specific antibody.

**Medium**

**LPS 20 μg/ml**

**αTLR4**

**αIgM**

**LPS-FITC**

**αTLR4 & αIgM**


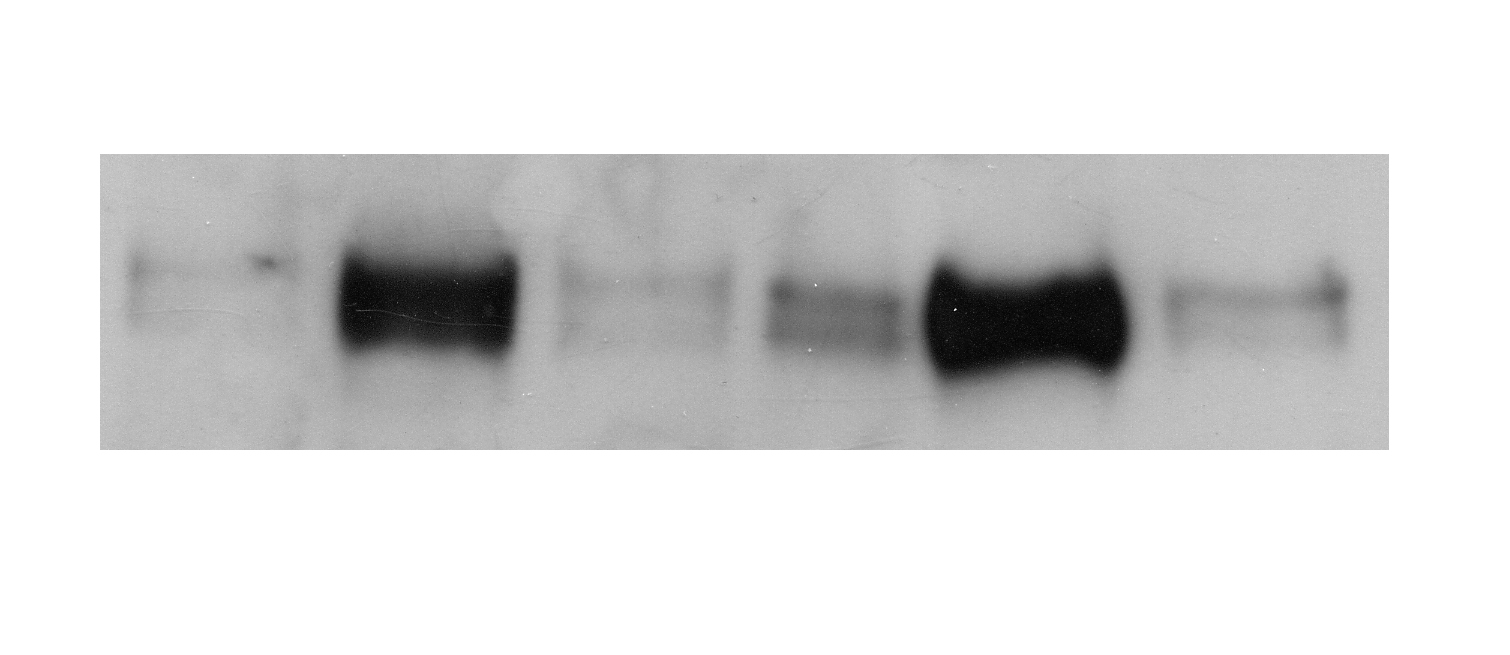


**αTLR-4**

**αIgM**

**ctrlAb**

**Medium**

**LPS**

**IP:**

**WB: αIgM**

**αTLR-4**

**αIgM**

**ctrlAb**

**B**

**A**
